# The PerioGene North study reveals that periodontal inflammation and advanced jawbone loss in periodontitis associate with serum gingipain antibodies but not with systemic autoimmunity

**DOI:** 10.3389/fimmu.2024.1504975

**Published:** 2025-01-14

**Authors:** Elin Kindstedt, Charlotte de Vries, Magnus Wänman, Barbara Aleksandra Potempa, Jan Potempa, Susanne Lindquist, Anders Esberg, Karin Lundberg, Pernilla Lundberg

**Affiliations:** ^1^ Department of Odontology, Section for Molecular Periodontology, Umeå University, Umeå, Sweden; ^2^ Wallenberg Centre for Molecular Medicine, Umeå University, Umeå, Sweden; ^3^ Division of Rheumatology, Department of Medicine Solna, Karolinska Institutet and Center for Molecular Medicine, Karolinska University Hospital, Stockholm, Sweden; ^4^ Department of Oral Immunology & Infectious Diseases, University of Louisville School of Dentistry, Louisville, KY, United States; ^5^ Department of Microbiology, Faculty of Biochemistry, Biophysics and Biotechnology, Jagiellonian University, Krakow, Poland; ^6^ LipumAB, Umeå, Sweden; ^7^ Department of Odontology, Umeå University, Umeå, Sweden

**Keywords:** periodontitis, alveolar bone loss, periodontal inflammation, cysteine peptidase gingipain B, anti-citrullinated protein antibodies

## Abstract

**Introduction:**

Periodontitis is associated with rheumatoid arthritis (RA). One hypothesis posits that this connection arises from the formation of autoantibodies against citrullinated proteins (ACPA) in inflamed gums, possibly triggered by *Porphyromonas gingivalis*. We previously demonstrated an increased antibody response to *P. gingivalis* arginine gingipains (anti-Rgp IgG), not only in individuals with severe periodontitis compared to controls, but in RA versus controls, with an association to ACPA. In the present study, we set out to further explore the relationship between anti-Rgp IgG, ACPA and periodontitis, including clinical periodontal parameters, in the large and well-characterized PerioGene North case-control study.

**Methods:**

We measured serum levels of anti-Rgp and ACPA IgG by enzyme-linked immunosorbent assay (ELISA), in 478 patients with periodontitis and 509 periodontally healthy controls within PerioGene North. Subsequently, anti-Rgp IgG levels and ACPA status were analysed in relation to periodontitis and clinical periodontal parameters.

**Results:**

Serum anti-Rgp IgG levels were elevated in cases versus controls (p< 0.001). However, receiver operating characteristic (ROC) curve analysis revealed that anti-Rgp IgG could not efficiently discriminate cases from controls (AUC= 0.63; 95% CI: 0.60 – 0.66). Among cases, increased anti-Rgp IgG levels associated with high periodontal inflammation and advanced alveolar bone loss (p<0.001 for both). An ACPA response was detected in 15 (3.1%) cases and 6 (1.2%) controls (p=0.033), but no association to periodontitis was evident after adjustment for age and smoking and anti-Rgp IgG levels did not differ between ACPA-positive and ACPA-negative individuals.

**Conclusion:**

We show that anti-Rgp IgG identifies a subgroup of periodontitis patients with high degree of periodontal inflammation and advanced alveolar bone loss, but we do not find support for a link between periodontitis or anti-Rgp IgG and ACPA status in PerioGene North. Given the association between anti-Rgp and alveolar bone loss, the mechanistic role of gingipains in bone resorption should be experimentally explored.

## 1 Introduction

Periodontitis is an oral condition that is characterized by progressive destruction of the tooth supporting tissues, including both collagenous connective tissue of the gingiva and the tooth anchoring jawbone (1). In periodontally healthy states, indigenous polymicrobial communities at mucosal surfaces maintain an ecological balance via inter-microbial and host-microbial interactions. However, genetic and acquired factors, most notably smoking, but also obesity, immune deficiencies, immunoregulatory defects, diabetes mellitus and other systemic diseases may disrupt this homeostatic balance, leading to selective growth of species with the potential for destructive inflammation. This condition, known as dysbiosis, underlies the development of periodontitis in susceptible hosts. The pathogenic process is not linear, but involves a positive-feedback loop between a dysbiotic microbiota on the tooth surface below the gum line and the host inflammatory response (2). The molecular pathways underpinning tissue destruction have not been clarified, and there are currently no biomarkers available to help clinicians predict onset of periodontitis or identify individuals at risk of a more aggressive disease course.

Besides the local and detrimental effects on the periodontium that jeopardize tooth retention, the disease is strongly connected to a number of other non-communicable diseases, including rheumatoid arthritis (RA) (3). Different models for a causal and reciprocal relationship between periodontitis and RA have been described (4). In support of a causal link between periodontitis and RA, results from experimental studies show that periodontitis can exacerbate arthritis (5). Our findings of increased alveolar bone loss in individuals with high levels of anti-citrullinated protein antibodies (ACPA), before RA onset, imply that periodontitis predates the onset of ACPA-positive RA (6). However, it remains elusive whether there is a causative link.

The gram-negative anaerobic oral bacterium *Porphyromonas gingivalis (P. gingivalis)* is frequently detected in subgingival plaque of periodontitis patients (7). *P. gingivalis* expresses multiple virulence factors for its growth, survival, and immune evasion (8), including the arginine gingipains (Rgp), potent extracellular cysteine proteases which efficiently degrade host proteins by cleaving polypeptides C-terminal of arginine (9, 10). Protein degradation by gingipains can facilitate further enzymatic processing by *Porphyromonas* peptidylarginine deaminase, resulting in citrullinated neoepitopes (11). Gingipains have also been described to contribute directly to the inflammatory response by cleavage of protease activated receptor-2 on the neutrophil surface, triggering formation of neutrophil extracellular traps with the release of endogenously citrullinated proteins (12). Notably, citrulline-reactive B cells have been successfully isolated from gingival tissue of periodontitis patients, suggesting that break of tolerance and ACPA production in RA may take place in inflamed gums (13). Moreover, gingipains are highly immunogenic (14), and we have previously shown an increased anti-Rgp antibody response, not only in individuals with periodontitis compared to controls, but also in RA, especially ACPA-positive RA, compared to controls even before RA onset (15, 16). We have previously demonstrated associations between anti-Rgp IgG and alveolar bone loss, as well as presence of ACPA in PAROKRANK, a case-control study including patients hospitalized for a first myocardial infarction. Interestingly, we also found a particularly strong association between anti-Rgp IgG and severe periodontitis in a subfraction of PerioGene North study (17). Therefore, *P. gingivalis* antibodies, more specifically gingipain antibodies, are gaining increased attention as a potential biomarker for individuals with periodontitis and concomitant risk for systemic autoimmunity (13, 16–18).

In summary, accumulating evidence suggests that periodontitis is linked to RA-specific autoimmunity through a loop in which *P. gingivalis* promotes ACPA formation. In the present study, we address this link by analysing the association between periodontal clinical parameters, anti-Rgp IgG and ACPA in 987 individuals within the large and well-characterized PerioGene North case-control study (19).

## 2 Material and methods

### 2.1 Study design

PerioGene North is a multicentre case-control study consisting of 526 periodontitis cases and 532 periodontally healthy controls. Study participants were consecutively recruited between 2007 and 2019, from specialist clinics and general dental care within the counties of Västerbotten, Gävleborg, Uppsala and Västmanland in northern Sweden. Cases were examined by senior consultants in periodontology and controls were examined by general dentists. To validate the absence of alveolar bone loss in controls, all radiographs were reviewed by senior consultants in periodontology.

### 2.2 Clinical data collection

All participants underwent a complete oral and periodontal examination, including registration of periodontal inflammation, measured as bleeding on probing (BoP), and periodontal probing pocket depth (PPD), at six sites per tooth using a PCP-12, 3-6-9-12 (Hu-Friedy) dental probe. Furcation involvement was assessed but not registered in the study protocol. Alveolar bone loss was assessed for each tooth using dental radiographs (bitewing and apical images). Information regarding number of teeth per quadrant with PPD (< 4 mm, 4-6 mm and > 6 mm) and degree of alveolar bone loss (< 1/3, ≥ 1/3 to ≤ 2/3 or > 2/3 of the root length) was registered in the study protocol. Data collection was conducted before the present classification for periodontitis was introduced (20). By using a proposed framework for applying the 2018 periodontal status classification on completed epidemiological studies, we concluded that all cases fulfilled the stage III criteria and 87 cases (18.2%) with <20 teeth could be classified as stage IV (21). This means that all cases presented with severe periodontitis. Cases were further subcategorized based on level of BoP (high ≥20%, low <20%), PPD and alveolar bone loss according to previous studies (22).

Degree of periodontal inflammation: A low degree of periodontal inflammation was defined as having BoP < 20%. High degree of periodontal inflammation was defined as having BoP ≥ 20%.

Level of periodontal pocket depth and alveolar bone loss: Each tooth was given a score of 1-3 depending on the PPD/alveolar bone loss value. A PPD < 4 mm or alveolar bone loss < 1/3 of the root length was given the score 1. A PPD between 4 and 6 mm or alveolar bone loss between ≥ 1/3 to ≤ 2/3 of the root length was given the score 2. A PPD > 6 mm or alveolar bone loss > 2/3 of the root length was given the score 3. The total score for the entire dentition was summed up and then divided by the number of teeth. In the present study, a score between 1.01 - 1.49 was referred to as a low degree of periodontal probing depth or alveolar bone loss, 1.50 – 1.99 as moderate, and over 2.0 as a high level.

Information about sex, birth country, past and/or current tobacco use, education level and awareness of parent with periodontitis (self-reported heredity) was recorded. Information about height and weight was obtained for calculation of body mass index (BMI). Information about general health (diseases according to ICD-10 categories) was obtained from registries of the National Board of Health and Welfare in Sweden.

### 2.3 Inclusion and exclusion criteria

Inclusion criteria for cases were i) having at least one tooth in each quadrant with alveolar bone loss ≥ 1/3 of the root length and ii) having ≥15 remaining teeth, or ≥8 if teeth were only present in one jaw. Cases with alveolar bone loss that could be explained by local aggravating factors such as root fractures or pulpal infections were excluded. The cases were included after being refered to specialist clinics from generel dental care and had received previous periodontal treatment to various extent. Inclusion criteria for controls were i) no alveolar bone loss, i.e. <3 mm distance from the cementoenamel junction to the bone crest, ii) PPD <4 mm, iii) having ≥24 remaining teeth and iv) being ≥ 34 years of age. Participants with known contagious blood diseases were excluded.

### 2.4 Blood sampling

A venous blood sample of 3 x 10 mL was collected from all participants at inclusion. The participants were not fasted at the time of sampling. Collection and handling of blood samples, including fractionation into plasma, serum, and buffy coat, and storage at -80°C followed the standardized routines at the Medical Biobank of Northern Sweden, Västerbotten County Council, Sweden.

### 2.5 Antibody measurements

Anti-Rgp IgG in serum were measured using a previously described *in-house* enzyme-linked immunosorbent assay (ELISA) (15, 17), with purified, recombinant hexahistidine-tagged RgpB protein as coating agent (23). To compare serum samples analysed on different ELISA plates, anti-Rgp IgG levels were presented as arbitrary units (AU) with interquartile ranges (IQR) calculated from a standard curve (a pool of anti-Rgp IgG positive sera in 1:1 x7 serial dilution) included on all plates. Serum samples were analysed in duplicates and blank wells were included on all plates to account for background signal.

Presence ACPA was measured as anti-cyclic citrullinated peptide2 (CCP2) IgG, using the Immunoscan CCPlus^®^ test kit (Svar Life Science, Malmö, Sweden), according to manufacturer’s instructions. Serum samples were analysed in single wells, with positive samples (≥25U/mL) re-analysed in duplicates.

### 2.6 Statistical methods

Descriptive analyses were used for frequency distributions of categorical variables, whereas median values with interquartile range were calculated for continuous variables. Group comparisons were conducted utilizing chi-square tests (categorical variables) or the non-parametric Mann-Whitney U test or Kruskal-Wallis test (continuous variables). Log-transformed anti-Rgp IgG levels were analyzed in relation to periodontitis, periodontal inflammation, periodontal pocket probing depth and alveolar bone loss, using a linear regression model, adjusted for age, age^2^, sex and smoking. Results are presented as Exp(B) = exponential B (interpretable as multiplicative effect), with 95% confidence interval (CI) and p-value. Adjustment for multiple testing was performed with Tukey’s method. P-values <0.05 were considered statistically significant. Receiver operating characteristic curves (ROC) was used to evaluate the discriminatory performance of anti-Rgp IgG to distinguish cases from controls and to determine the area under the curve (AUC). Youden’s J statistic was used to determine the cutoff with highest sensitivity and specificity.

Statistical Package for Social Sciences (SPSS), version 26 (IBM Corporation, Armonk, NY, USA), and R, version 4.2.3 (R foundation for statistical computing, Vienna, Austria), with packages: *tidyverse, magrittr, haven, psych, cutpointr, pRoc, emmeans, gtsummary*, and *brglm2*, were used for the analyses.

## 3 Results

### 3.1 Study population characteristics

Among 1,058 enrolled study participants, 60 were excluded in the present study due to missing clinical data or missing serum, and eleven were excluded due to not fulfilling the inclusion criteria, resulting in 478 study participants with periodontitis (referred to as cases) and 509 periodontally healthy (referred to as controls). The distribution of men and women was similar between cases and controls, but cases were older and had a higher proportion of smokers. Two controls and three cases were diagnosed with rheumatoid arthritis (**Table 1**). A comprehensive description of the study population characteristics has been published previously (19).

**Table 1:** Study participant characteristics in PerioGene North.

Baseline characteristics	Control (n=509)	Case (n=478)	Total (n=987)	*P*-value
Age, median (IQR)	44.0 (39.0-50.0)	59.0 (49.0-66.0)	50.0 (41.0-60.0)	< 0.001
Sex (%)				
Male	218 (42.8)	198 (41.4)	416 (42.1)	0.699
Female	291 (57.2)	280 (58.6)	571 (57.9)	
Smokers^a^, *n* (%)				
No	404 (79.4)	120 (25.1)	524 (53.1)	< 0.001
Yes	105 (20.6)	358 (74.9)	463 (46.9)	
Periodontal parameters				
Number of teeth	28 (27-28)	24 (21-27)	27 (24-28)	< 0.001
BoP %, median (IQR)	5 (1-12)	25 (13-44)	12 (4-31)	< 0.001
BoP level^b^ (%)				
Low (< 20%)	N/A	185 (38.7)	N/A	
High (≥ 20%)	N/A	291 (60.9)	N/A	
PPD level^b^ (%)				
None	N/A	7 (1.5)	N/A	
Low	N/A	75 (15.7)	N/A	
Moderate	N/A	193 (40.4)	N/A	
High	N/A	203 (42.5)	N/A	
Alveolar bone loss level^b^ (%)				
Low	N/A	117 (24.5)	N/A	
Moderate	N/A	246 (51.5)	N/A	
High	N/A	115 (24.1)	N/A	
RA status and antibody levels				
Rheumatoid arthritis (%) *(ICD-10 code M05-06)*	2 (0.4)	3 (0.6)	5 (0.5)	0.604
anti-Rgp IgG, median AU (IQR)	154.2 (79.4-300.8)	237.2 (125.3-611.9)	191.8 (98.5-402.4)	< 0.001
ACPA+, *n* (%)	6 (1.2)	15 (3.1)	21 (2.1)	0.033

IQR, interquartile range; BoP, bleeding on probing; PPD, pocket probing depth; AU, arbitrary units; N/A, not applicable as only cases were subcategorized according to BoP, PPD and alveolar bone loss.

a) Smokers include current and former smokers.

b) Subcategorization of periodontal parameters within the case group. Group categorization was based on a PPD/alveolar bone loss score. None = ≤ 1.00, low = 1.01-1.49, moderate = 1.50 – 1.99, high = ≥ 2.00. The score was calculated by assigning each tooth a score of 1-3 depending on the degree of PPD/alveolar bone loss. The total score for the entire dentition was summed and then divided by the number of teeth.

### 3.2 High anti-Rgp IgG levels are associated with periodontitis, but show poor potential to discriminate between cases and controls

**Figure 1:**
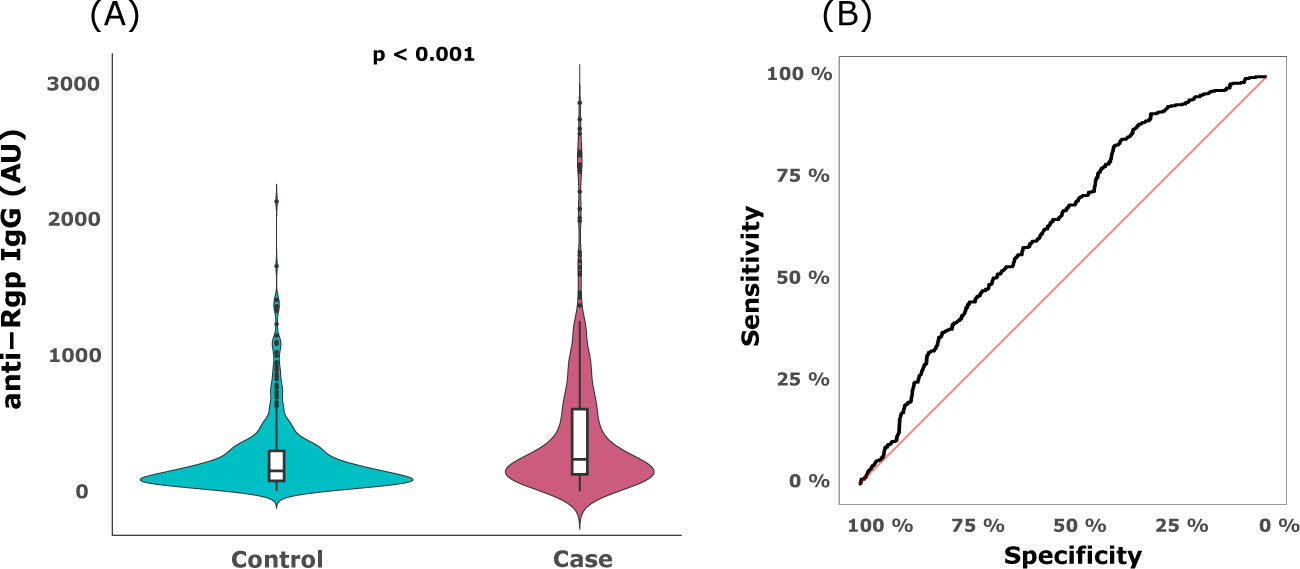
Serum anti-Rgp IgG levels are elevated in periodontitis, but do not discriminate cases from controls. **(A)** The violin plot shows distribution of serum anti-Rgp IgG levels in PerioGene North cases (n=478) and controls (n=509). Integrated box plots show median AU values and 25^th^ and 75^th^ percentiles as horizontal lines; whiskers indicate the 10^th^ to 90^th^ percentile; outliers plotted as dots. **(B)** Receiver operating characteristic (ROC) curve for prediction of cases and controls based on serum levels of anti-Rgp IgG. Rgp = arginine gingipain; AU = arbitrary units.

An antibody response towards *P.gingivalis* Rgp was evident in both periodontitis cases and controls, with higher median anti-Rgp IgG values for cases (237.2 AU; IQR= 125.3-611.9) than controls (154.2 AU; IQR= 79.4-300.8), p<0.001 (**Table 1** and **Figure 1A**). The average increase of anti-Rgp IgG levels in cases versus controls was 71% (p<0.001) when adjusted for age, age^2^, sex and smoking (**Table 2**). A ROC curve analysis showed that anti-Rgp IgG levels could separate cases from controls with a sensitivity of 37.5% and specificity of 83.1% (AUC=0.63; 95% CI: 0.60 – 0.66), demonstrating a weak discriminatory ability (**Figure 1B**).

**Table 2:** Association between serum anti-Rgp IgG and periodontitis.

	Anti-Rgp IgG level	*P-value*
	*Exp(B) (95% CI)*	
Category		
Control	Reference	
Case	1.71 (1.45-2.02)	< 0.001
Covariate		
Sex		
Men	Reference	
Women	0.92 (0.81-1.11)	0.226
Smoker^a^		
No	Reference	
Yes	0.92 (0.79-1.08)	0.302
Age	0.98 (0.93-1.03)	0.347
Age^2^	1.00 (1.00-1.00)	0.375

Anti-Rgp IgG levels were analyzed in relation to periodontitis using a linear regression model. Age, age^2^, sex and smoking were included in the model as covariates. B = regression coefficient; Exp(B) = exponential B (interpretable as multiplicative effect); CI = confidence interval. ^a^Smokers includes current and former smokers.

### 3.3 Anti-Rgp IgG levels associate with periodontal inflammation and alveolar bone loss

**Figure 2:**
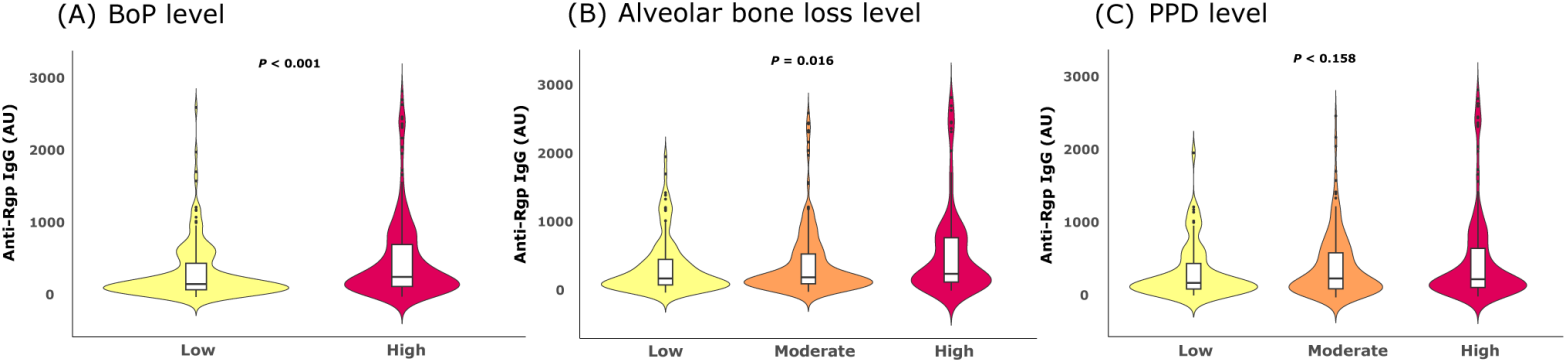
Anti-Rgp IgG levels are elevated in cases with periodontal inflammation and advanced alveolar bone loss. Violin plots show distribution of Rgp IgG levels in PerioGene North cases, based on: **(A)** degree of periodontal inflammation, measured as bleeding on probing (BoP); **(B)** level of alveolar bone loss and **(C)** periodontal pocket probing depth (PPD) level. Integrated box plots show median AU values and 25^th^ and 75^th^ percentiles as horizontal lines; whiskers indicate 10^th^ to 90^th^ percentile; outliers are plotted as dots. Rgp = arginine gingipain; AU = arbitrary units.

Next, we analyzed anti-Rgp IgG levels in relation to different clinical periodontal parameters; periodontal inflammation, measured as bleeding on probing, (BoP), periodontal probing pocket depth (PPD), and alveolar bone loss. We detected significantly higher anti-Rgp IgG levels in cases with high periodontal inflammation (**Figure 2A**). The average increase in anti-Rgp IgG levels in cases with high versus low periodontal inflammation was 50%, p<0.001 (**Table 3**). We also found higher anti-Rgp IgG levels in cases with high level of alveolar bone loss (**Figure 2B**). For cases with high versus low level of alveolar bone loss, the average increase of anti-Rgp IgG levels was 65%, p<0.002 (**Table 3**), and here we also observed a significant increase (40%) when comparing cases with high versus moderate level of alveolar bone loss, p=0.042. We found no association between anti-Rgp IgG levels and level of PPD (**Figure 2C**, **Table 3**). These analyses were adjusted for the confounding effects of age, age^2^, sex and smoking.

**Table 3:** Average increase in anti-Rgp IgG levels between different clinical subgroups of periodontitis cases.

Cases								
	BoP (level)		PPD (level)		Alveolar bone loss (level)			
	*Exp(B)* *(95%CI)*	*P-value*	*Exp(B)* *(95%CI)*	*p-value*	*Exp(B)* *(95%CI)*			*p-value*
Category								
Low	Reference							
Moderate	N/A	N/A	1.18 (0.88-1.59)	0.262	1.22 (0.92-1.64)		0.228	
High	1.50 (1.22-1.83)	< 0.001	1.32 (0.98-1.76)	0.064	1.65 (1.17-2.33)		0.002	
Moderate vs High	N/A	N/A	1,12 (0.84-1.48)	0.749	1.4 (1.0-1.8)		0.0417	
Covariate								
Sex								
Men	Reference							
Women	0.82 (0.67-1.00)	0.048	0.82 (0.67-1.00)	0.055	0.81 (0.67-0.99)	0.044		
Smoker^a^								
No	Reference							
Yes	0.75 (0.60-0.94)	0.015	0.73 (0.58-0.92)	0.008	0.72 (0.57-0.91)	0.006		
Age	0.99 (0.93-1.05)	0.745	0.98 (0.92-1.04)	0.479	2.43 (0.28-20.9)	0.418		
Age^2^	1.00 (1.00-1.00)	0.616	1.00 (1.00-1.00)	0.407	3.47 (0.40-29.7)	0.257		

a) Smokers include current and former smokers.

Anti-Rgp IgG levels were analyzed in relation to periodontitis subcategories using a linear regression model. Age, age^2^, sex and smoking were included in the model as covariates. Comparisons between BoP level is only applicable between low and high groups since it is a dichotomous variable. B = regression coefficient; Exp(B) = exponential B (interpretable as multiplicative effect); CI, confidence interval; BoP, bleeding on probing; PPD, pocket probing depth; N/A, not applicable.

### 3.4 RA-specific autoimmunity does not associate with periodontitis or anti-Rgp IgG

Next, we analyzed the presence of ACPA IgG in PerioGene North. When applying the manufacturer’s suggested cutoff value of ≥25 U/mL, 21 individuals were considered ACPA-positive. Notably, 15 of these had periodontitis, giving a frequency of 3.1% ACPA-positive cases and 1.2% ACPA-positive controls, p=0.003 (**Table 1**). Among the ACPA-positive participants, 1 case and 2 controls had been diagnosed with RA. Yet, there was no significant association between periodontitis and ACPA after adjusting for age and smoking (data not shown). Furthermore, anti-Rgp IgG levels did not differ significantly between ACPA-positive and ACPA-negative individuals (data not shown).

## 4 Discussion

In this study, we demonstrate that serum anti-Rgp IgG levels cannot clearly discriminate individuals with periodontitis from periodontally healthy but define a subgroup of periodontitis patients with high periodontal inflammation and advanced alveolar bone loss. Moreover, we find no strong support for a link between periodontitis or anti-Rgp IgG and RA-specific autoimmunity, e.g. ACPA.

Antibodies against periodontal pathogens have been investigated for many years (24). In particular, antibodies against *P. gingivalis* have been shown to associate with periodontitis (25), and among 29 different *P. gingivalis* antigens, Hirai and co-authors recently showed that the gingipains elicited the most sensitive and specific IgG responses in patients with periodontitis (14), supporting the use of Rgp in our study.

The data presented herein clearly show that individuals with periodontitis present with higher anti-Rgp IgG levels than periodontally healthy controls, which is in accordance with our previous findings (17). It should be noted that high anti-Rgp IgG levels were also detected among some of the controls. Studies have suggested that periodontally healthy individuals are able to produce highly functional antibodies, protecting against *P. gingivalis* colonization, whereas periodontitis patients produce less functional antibodies with lower avidity (26). Hence, we speculate that the presence of anti-Rgp antibodies in periodontally healthy controls in our study reflects a healthy immune response, while high levels of anti-Rgp IgG that we see in a subset of periodontitis patients indicate high *P. gingivalis* counts and high pathogenic gingipain load. In support of this scenario, several studies showed decreased anti-*P. gingivalis* antibody levels in response to successful periodontal treatment, following lower bacterial counts (27–29).

Our previous data (17), based on 41 cases and 39 controls from PerioGene North, indicated a very good capacity for anti-Rgp antibodies to discriminate cases from controls (AUC=0.79). Thus, we wanted to further validate our data in the entire cohort. However, in the present study, anti-Rgp IgG had poor discriminatory ability to distinguish cases from controls (AUC=0.63). The discrepant data between the two studies suggests that the sub-cohort is unrepresentative of the entire PerioGene North cohort. Moreover, the results in the present study are in agreement with our previous findings in PAROKRANK, a case-control study designed to investigate periodontal health in relation to myocardial infarction, where anti-Rgp IgG also could not separate periodontitis patients from controls efficiently, but was still significantly elevated in individuals with severe forms of periodontitis compared to controls (17).

In here, we present significant associations between anti-Rgp IgG and specific clinical periodontal parameters, periodontal inflammation and alveolar bone loss. Thus, collectively, these data indicate that anti-Rgp antibodies are not likely to perform well as predictive biomarkers for periodontitis *per se*, while they may still be valuable indicators of risk for severe periodontal damage.

Gingipains are known for their proteolytic capacity, including the degradation of components of the complement system and extracellular matrix structures (30). They can directly contribute to gingival bleeding and tissue breakdown by degrading fibrinogen (31). Interestingly, gingipains have also been shown to promote osteoclastogenesis *in vitro*, thus implicating a possible role in bone resorption (32). Noteworthy, both gingipain vaccines and gingipain-inhibitors have been used successfully to prevent *P. gingivalis*-induced periodontal damage in experimental models (33, 34). Moreover, we have previously shown elevated anti-Rgp antibody levels in RA versus controls (15), and given the results in the present study – demonstrating an association between high anti-Rgp antibody levels and advanced alveolar bone loss – anti-Rgp IgG levels should potentially also be examined in relation to articular bone loss in RA. Notably, a recent study links oral bacteremia to RA flares (35), and other studies have detected *P. gingivalis* DNA in RA synovial joints (36, 37). Thus, the mechanistic role of gingipains in inflammation-mediated bone resorption should be further explored.

Several studies have linked periodontitis and periodontal pathogens to ACPA-positive RA (15, 35, 38), including a recently published meta-analysis demonstrating that RA patients with periodontitis have significantly higher ACPA levels than RA patients without periodontitis (39). However, until now, it has not been clear if periodontitis alone is sufficient to elicit a systemic ACPA response, as previous studies are sparse and present conflicting results. The present study is, to the best our knowledge, the first to investigate ACPA status in a large and well-characterized periodontitis case-control study. Although we observed a higher frequency of ACPA-positivity among periodontitis patients compared to controls, in line with our previous report (17), this was not significant after adjustment for the confounding effects of smoking and age. Given that only 15 of 478 individuals with periodontitis were ACPA-positive, we were underpowered to analyze ACPA in relation to periodontal parameters.

In accordance with the data herein, a cross-sectional study by Lew and co-authors did not find differences in ACPA levels when comparing periodontitis patients without RA and healthy controls (40). In here, we did not find an association between ACPA status and anti-Rgp. Similarly, Svard et al. found no association between anti-Rgp antibodies in saliva and periodontitis or ACPA status (41). In summary, the data we present are based on a large and well-characterized periodontitis cohort and suggest that there is not convincing evidence for that a systemic ACPA response is connected to periodontitis, or elevated anti-Rgp IgG levels in periodontitis patients. Still, this does not rule out that locally produced ACPA in inflamed gingival tissue are involved in onset and/or progression of RA in susceptible individuals. Notably, ACPA have been detected in gingival crevicular fluid from non-RA individuals (42).

The main strength with the present study is the significant power of PerioGene North, owing to its large number of study participants and the high resolution clinical periodontal data provided by trained specialists. Within this material, we have previously described serum levels of hs-CRP that are in line with results presented in a meta-analysis (43), implying that PerioGene North is well suited for investigating, and detecting, serum markers associated with periodontitis. Unlike the periodontitis cases in PAROKRANK, where more than half were affected by myocardial infarction, the overall presence of general diseases in PerioGene North is low (19). Therefore, the elevated anti-Rgp IgG levels observed in PerioGene North are less likely to be affected by the presence of other systemic diseases.

A limitation with our study was that the case group, due to the original purpose of PerioGene North to study genetic polymorphisms, were not matched to controls on age. However, all controls were 34 years or older, which is a strength as many other studies have included young adults as periodontally healthy controls. Still, this resulted in an older case group and all analyses were subsequently adjusted for age as well as for smoking, both well-known risk factors for periodontitis (44, 45). In our previous report (19), *post-hoc* analysis within different age groups confirmed that the distinguishable protein profile was not a result of the case group being older.

In conclusion, our data show that periodontitis *per se* is not associated with the presence of a systemic ACPA response and that ACPA-positive individuals in this cohort do not have an increased antibody response to *Pg* virulence factor arginine gingipain. While anti-Rgp IgG showed poor ability to separate patients with periodontitis from periodontally healthy in an efficient manner, elevated levels associate significantly with the subset of patients that have active periodontal inflammation and advanced alveolar bone loss. Prospective studies could reveal whether high anti-Rgp IgG levels can serve as a biomarker to predict a more aggressive disease course. Future studies should also address the actions of gingipains in the context of bone resorption, to clarify molecular mechanisms, and further explore whether blocking gingipains could be a future treatment for periodontitis.

## Data Availability

The original contributions presented in the study are included in the article/supplementary material. Further inquiries can be directed to the corresponding author.
